# Multimodal prehabilitation to reduce the incidence of delirium and other adverse events in elderly patients undergoing elective major abdominal surgery: An uncontrolled before-and-after study

**DOI:** 10.1371/journal.pone.0218152

**Published:** 2019-06-13

**Authors:** T. L. Janssen, E. W. Steyerberg, J. C. M. Langenberg, C. C. H. A. van Hoof- de Lepper, D. Wielders, T. C. J. Seerden, D. C. de Lange, J. H. Wijsman, G. H. Ho, P. D. Gobardhan, R. van Alphen, L. van der Laan

**Affiliations:** 1 Department of Surgery, Amphia Hospital, Breda, the Netherlands; 2 Department of Public Health, Erasmus MC–University Medical Center Rotterdam, Rotterdam, the Netherlands; 3 Department of Gastroenterology, Amphia Hospital, Breda, the Netherlands; 4 Department of Geriatrics, Amphia Hospital, Breda, the Netherlands; 5 Department of Physical Therapy, Amphia Hospital, Breda, the Netherlands; University of Glasgow, UNITED KINGDOM

## Abstract

**Background:**

Delirium is a common and serious complication in elderly patients undergoing major abdominal surgery, with significant adverse outcomes. Successful strategies or therapies to reduce the incidence of delirium are scarce. The objective of this study was to assess the role of prehabilitation in reducing the incidence of delirium in elderly patients.

**Methods:**

A single-center uncontrolled before-and-after study was conducted, including patients aged 70 years or older who underwent elective abdominal surgery for colorectal carcinoma or an abdominal aortic aneurysm between January 2013 and October 2015 (control group) and between November 2015 and June 2018 (prehabilitation group). The prehabilitation group received interventions to improve patients’ physical health, nutritional status, factors of frailty and preoperative anaemia prior to surgery. The primary outcome was incidence of delirium, diagnosed with the DSM-V criteria or the confusion assessment method. Secondary outcomes were additional complications, length of stay, unplanned ICU admission, length of ICU stay, readmission rate, institutionalization, and in-hospital or 30-day mortality.

**Result:**

A total of 360 control patients and 267 prehabilitation patients were included in the final analysis. The mean number of prehabilitation days was 39 days. The prehabilitation group had a higher burden of comorbidities and was more physically and visually impaired at baseline. At adjusted logistic regression analysis, delirium incidence was reduced significantly from 11.7 to 8.2% (OR 0.56; 95% CI 0.32–0.98; P = 0.043). No statistically significant effects were seen on secondary outcomes.

**Conclusion:**

Current prehabilitation program is feasible and safe, and can reduce delirium incidence in elderly patients undergoing elective major abdominal surgery. This program merits further evaluation.

**Trial registration:**

Dutch Trial Registration, NTR5932.

## Introduction

Our society is ageing progressively. The number of people above 60 years old in the world is expected to more than double in the next 35 years [1]. Incidence rates of common age-related diseases, such as colorectal carcinoma and abdominal aortic aneurysms, that require surgery are likely to undergo a similar increase [2, 3]. Elderly people undergoing surgery for these conditions have an increased risk of postoperative complications and mortality [4, 5]. Roughly 30% of the patients that undergo a colorectal resection develop postoperative complications [6], which leads to adverse outcomes and a decrease in quality of life in elderly patients [7, 8].

With progressive ageing, a person’s physical resilience decreases while frailty increases [9]. Up to forty-three percent of colorectal cancer patients can be considered frail [10], which makes them likely to be unable to withstand physical stress associated with surgery [11]. The risk for morbidity, mortality and institutionalization is increased over four times in these patients [12, 13]. A recent meta-analysis showed an independent relationship between frailty and delirium, a common and serious postoperative complication in elderly surgical patients [14].

Despite advances in techniques such as minimally invasive surgery and enhanced recovery after surgery (ERAS) protocols, recent incidence rates of postoperative delirium vary from 4–35% in CRC patients [15–21] and from 13–29% in AAA patients [15, 22, 23]. Delirium is a major complication leading to a prolonged length of hospital stay (LOS), increased healthcare costs, institutionalization, decreased quality of life and increased morbidity and mortality [14, 15, 21, 24–27].

Delirium is defined as a neuropsychiatric disorder, caused by a combination of predisposing and precipitating risk factors. Baseline vulnerability is set by predisposing risk factors, such as old age, frailty, comorbidities and functional, cognitive and sensory impairment [24, 28, 29]. An additional risk for delirium is added by precipitating risk factors, such as nutritional impairment, polypharmacy, anaemia, pain, and type of surgery [24, 28, 29]. Patients with higher baseline vulnerability need fewer precipitating factors to develop a delirium.

A recent meta-analysis concluded that there was strong evidence for multicomponent interventions to prevent delirium in hospitalised non-ICU patients [30]. The multifactorial aetiology of delirium is exemplified by the evidence that delirium incidence in elderly patients can be reduced by optimizing multiple precipitating risk factors at once [30, 31]. Examples of such efforts, implemented during admission, are the Hospital Elder Life Program (HELP) and the NICE guidelines [31, 32].

To further reduce the number of postoperative adverse events, prehabilitation programs were investigated in recent studies. Prehabilitation is the optimization of patients’ physical, nutritional and psychological health before admission, in order to withstand the stress caused by surgery and prevent postoperative adverse events [33]. Recent reviews on prehabilitation programs did not reach a consensus on efficacy and labelled the quality of the majority of these studies as poor [34–37]. Studies implementing these programs are heterogeneous and focussed mainly on colorectal cancer patients, while only two studies focussed on patients undergoing vascular surgery [35, 37].

In the case of elective surgery, an additional preventive effect on delirium might be achieved by adding such a prehabilitation program to already existing ERAS protocols and multicomponent intervention programs. That way, elements of frailty, poor preoperative fitness and malnutrition can be identified and tackled even in the pre-admission period. The aim of this study was to reduce the incidence of delirium and other postoperative adverse events by prehabilitating CRC and AAA patients prior to surgery. The focus of this prehabilitation program is to optimize patients’ physical health and nutritional status, to tackle factors of frailty and to correct preoperative anaemia before admission.

## Methods

### Study design, setting and participants

This study is a single-center, uncontrolled before-and-after study, conducted in the Amphia hospital in Breda, the Netherlands. Patients aged 70 or older who underwent elective abdominal surgery for CRC or AAA were included.

Past studies on delirium have likewise used a combination of diseases in their trials [27, 38, 39]. CRC and AAA were chosen for this trial because these are common conditions in the elderly, with relatively high numbers of postoperative complications and both require major abdominal surgery.

Patients were considered ineligible when acute hospitalization or surgery was required, when they had surgery six months prior to diagnosis or when surgery was planned within 2 weeks of the multidisciplinary meeting. The control group consisted of a historical population meeting the same inclusion and exclusion criteria as the prehabilitation group and underwent surgery between January 2013 and October 2015. The prehabilitation group was formed by patients who followed the prehabilitation program and were operated on between November 2015 and June 2018. Eligibility was assessed and optimal treatment was determined during colorectal and vascular multidisciplinary meetings. Treatment options for CRC patients were (robot-assisted) laparoscopic or open tumour resection. For AAA patients, options were open or (fenestrated) endovascular aortic repair. Written informed consent was obtained during trial enrolment, before the first outpatient clinic visit.

A multidisciplinary care pathway was designed, as an addition to already existing care interventions, to optimize patients’ physical and nutritional health and factors of frailty prior to admission. All prospective patients followed this prehabilitation pathway for an optimal period of 5 weeks prior to surgery. The control group was not prehabilitated. For both patient groups, preventive measures for delirium were taken during admission according to the HELP guidelines [31] and postoperative patient care was provided according to ERAS protocols. Due to the design of this study, no randomization or blinding of the persons involved in this research was possible. The study design and methods have been described in detail previously [40]. An overview of the complete study period and design is added to this paper (S1 Table). This trial has been previously registered in the Dutch Trial Registration, with trial number NTR5932. Initially, the medical ethical committee decided registration of this trial was not required. However, following guidelines of good clinical practice, this trial was retrospectively registered six months after commencement of the trial.

### Intervention

During the outpatient clinic visit, a nurse practitioner and a physiotherapist performed a complete assessment of patients’ basic health, fitness and factors of frailty. A dietician was consulted in case of undernourishment, decreased appetite or unintentional weight loss. A complete overview of the interventions that were performed has previously been published [40].

A trained nurse practitioner collected baseline characteristics and screened for factors of frailty. Delirium risk was assessed by collecting information on cognitive impairment, sensory impairment, functional dependency, and burden of comorbidity (using Charlson Comorbidity Index (CCI) and American Society of Anesthesiologists (ASA) score) [41, 42]. KATZ-ADL and SNAQ were scored to objectively assess physical dependence and nutritional impairment respectively [43, 44]. In case of a patient being a smoker, the nurse practitioner emphasized the importance of smoking cessation.

A physiotherapist provided patients with home-based personalised exercise programs, depending on a patient’s capabilities, to increase respiratory muscle strength, muscle strength of both legs and overall fitness. These exercises consisted of aerobic training, resistance training and respiratory muscle training. To prevent an even further increase of the physical and mental burden of this research on the patient, improvements in fitness were not quantified preoperatively during admission. However, previous research has shown evidence of improvement in functional capacity when patients performed similar, unsupervised home-based exercises [35]. Patients were asked to keep a diary with a record of their daily activities to assess compliance with the prehabilitation program. When the diary was not kept, data on compliance was obtained through telephone contact. Patients were considered non-compliant when compliance could not be assessed or when patients performed less than 75% of provided exercises.

A dietician objectively quantified nutritional status using body mass index, blood levels of pre-albumin and vitamins B and D, and the mini nutritional assessment short form (MNA-SF) [45]. Patients were given dietary instructions based on a minimum daily protein intake of 1.2 grams per kilogram bodyweight and a caloric intake of a patient’s basal need plus 30%. Supplemental protein drinks were provided when daily intake was not sufficient.

If indicated, patients subsequently visited a geriatrician, who performed a Complete Geriatric Assessment [46]. The geriatrician assessed the need for supplementary interventions to prevent delirium during admission and provided the surgical wards with advice on which additional preventive measures to use (e.g. prescribing prophylactic haloperidol, critically reviewing medication, and providing advice regarding prevention of infection, falls, pain, anxiety and dehydration).

Anaemic patients (haemoglobin level of < 7.4 mmol/L(<120 g/L) for women and < 8.1 mmol/L for men (<130 g/L)) received an intravenous iron injection during admission at day-care to increase preoperative haemoglobin levels [47].

### Outcomes

The primary outcome was incidence of delirium. Ward nurses screened for delirium during regular rounds using the delirium observation screening scale [48, 49]. This scale is a validated and easy-to-use delirium screening tool for nurses with a sensitivity of 94% and a specificity of 78% [50]. When delirium was suspected, a geriatrician was consulted to confirm the diagnosis using the DSM-V criteria or the Confusion Assessment Method [51, 52]. Secondary outcomes were length of hospital stay (LOS), unplanned ICU admission, length of ICU stay, readmission rate, additional in-hospital complications, discharge disposition, and in-hospital or 30-day mortality.

### Statistical analysis

The sample size was calculated based on data from a previous study [15]. Based on this analysis, a 50–50 trial needed 550 patients, or 275 patients per study arm, to gain an absolute risk reduction of the incidence of delirium of 7.5%. These calculations are based on a power of 80% with a 5% two-sided significance level.

Dichotomous variables were presented as frequencies with percentages and continuous variables as medians with interquartile range. Differences in these characteristics were tested for statistical significance by using Pearson chi-squared test or Fisher’s Exact test and Student t-test or Mann-Whitney U test respectively, depending on the distribution of the data.

Unadjusted and adjusted regression analyses were performed to calculate odds ratios (OR) and 95% confidence intervals (CI) for the primary and secondary outcomes. In the multivariate regression analysis, the primary outcome was adjusted for important predisposing covariates (age, history of delirium and ASA ≥3), as found in earlier research [15]. For our secondary outcomes, history of delirium was replaced with type of surgery (EVAR, open AAA, laparoscopic or open CRC) as covariate for the multivariate regression analysis [53, 54]. Linear regression analysis and logistic regression analysis were performed in case of continuous and dichotomous outcome variables respectively.

All data was gathered using the electronic patient file ‘Hyperspace Version IU4 (Epic, Inc., Verona, WI)’. Data for the control group was prospectively stored in the electronic patient files and retrospectively obtained. Statistical analyses were performed using IBM SPSS statistics software (SPSS Inc., Chicago, Illinois, USA) and ‘R’ statistical software version 3.5.1 (The R Project for Statistical Computing) with the ‘forest plot’ package. A two-sided p-value <0.05 was considered statistically significant. Missing data was not imputed.

This article has been reported according to the CONSORT guidelines [55].

## Results

**Fig 1:**
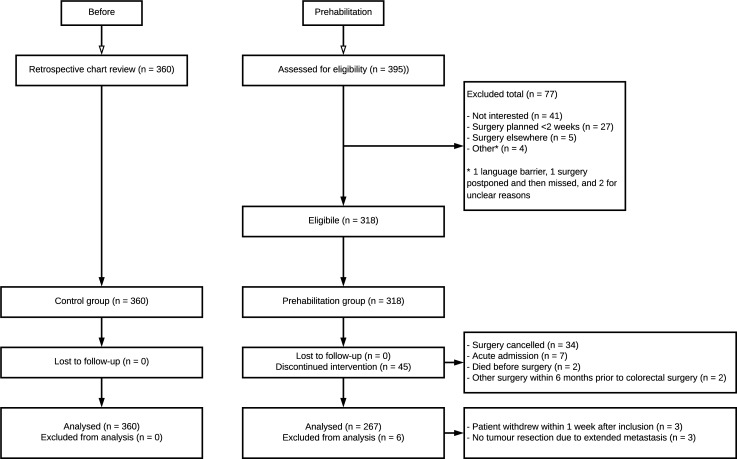
CONSORT flow diagram.

For the control group, 360 patients were considered eligible. In our prehabilitation group, 395 prospective patients were assessed for eligibility. A total of seventy-seven patients were excluded, mostly due to not being interested or early planning of surgery. Of the 318 eligible patients that entered the prehabilitation program and were seen in the outpatient clinic, 45 patients discontinued intervention and 6 were excluded from further analysis, leaving a total of 267 patients who were included in the final analysis. Two patients died before surgery, one due to respiratory failure and one due to a cerebral infarction. Fig 1 shows a diagram with a complete overview of eligibility, patient allocation and attrition [55].

The mean number of prehabilitation days, the number of days from the outpatient clinic visit to surgery, was 39 days (95% CI; 35–43 days). The time between the multidisciplinary meeting and surgery was less than 14 days in four patients, however due to the intention-to-treat design of our study they were still included in our analysis.

### Patient characteristics

Table 1 presents full sample baseline characteristics and baseline characteristics per diagnosis of both the control group and the prehabilitation group. A complete overview of baseline comorbidities is added to this paper (S2 Table). Significantly more patients in the prehabilitation group had a higher burden of comorbidity and were physically and visually impaired. Intravenous iron was given to 103 anaemic patients, of which 84.5% were CRC patients.

**Table 1:** Full sample baseline characteristics and baseline characteristics per diagnosis in control group and prehabilitation group.

	Control group N = 360		Prehabilitation group N = 267		Full sample (N = 627)		
	AAA N = 73 (20.3%)	Colorectal cancer N = 287 (79.7%)	AAA N = 70 (26.2%)	Colorectal cancer N = 197 (73.8%)	Controls N = 360 (%)	Prehabilitation N = 267 (%)	P-value^a^
Age, median (IQR)	75 (72–80)	76 (73–80)	76.5 (72–80)	77 (74–81.5)	76 (73–80)	77 (73–81)	0.15
Male gender	63 (86.3)	162 (56.4)	59 (84.3)	114 (57.9)	225 (62.5)	173 (64.8)	0.56
**(Burden of) comorbidities**							
Cognitive impairment	3 (4.1)	22 (7.7)	1 (1.4)	18 (9.1)	25 (6.9)	19 (7.1)	0.93
History of delirium	2 (2.7)	17 (5.9)	5 (7.1)	12 (6.1)	19 (5.3)	17 (6.4)	0.56
CCI^b^ ≥ 7	18 (24.7)	95 (33.1)	27 (38.6)	83 (42.1)	113 (31.4)	110 (41.2)	0.011
ASA^b^ score ≥ 3	47 (64.4)	101 (35.2)	51 (72.9)	98 (49.7)	148 (41.1)	149 (55.8)	<0.001
**Physical impairment**								
KATZ-ADL score ≤5	6 (8.2)	41 (14.3)	13 (18.6)	46 (23.4)	47 (13.1)	59 (22.1)	0.003
**Nutritional status**^c^							
SNAQ score ≥ 3	6 (8.2)	71 (24.7)	4 (5.7)	42 (21.3)	77 (21.4)	46 (17.2)	0.20
**Intoxications**							
Daily alcohol use^c^	27 (37.0)	109 (38.0)	26 (37.1)	82 (41.6)	136 (37.8)	108 (40.4)	0.50
Active smoker^d^	22 (31.4)	35 (12.5)	19 (27.1)	25 (12.7)	57 (16.2)	44 (16.5)	0.94
**Sensory impairment**						
Visual impairment	16 (21.9)	80 (27.9)	27 (38.6)	80 (40.6)	96 (26.7)	107 (40.1)	<0.001
Hearing impairment	24 (32.9)	88 (30.7)	25 (35.7)	62 (31.5)	112 (31.1)	87 (32.6)	0.70
**Surgery**							
Open surgery	27 (37.0)	88 (30.7)	18 (25.7)	23 (11.7)	115 (31.9)	41 (15.4)	<0.001
Minimally invasive surgery	46 (63.0)	199 (69.3)	52 (74.3)	174 (88.3)	245 (68.1)	226 (84.6)	

a: Calculated for full samples of control versus prehabilitation

b: CCI: Charlson Comorbidity Index; ASA: American Society of Anesthesiologists

c: <3% of retrospective data missing

d: <10% of retrospective data missing

### Compliance

The overall compliance to the prehabilitation program was 73.9%. Muscle-strength exercises were performed according to protocol by 74.2% of all patients. For daily walking and breathing exercises, rates were 75.7% and 71.9% respectively.

### Outcomes

The short-term postsurgical outcomes are presented in Table 2. Unadjusted and adjusted odds ratios (OR) with 95% confidence intervals for primary and secondary outcomes are presented in Table 3. Incidence of delirium was decreased by almost a third, from 11.7% to 8.2% (P = 0.16). After adjustment for important prognostic confounders, the prehabilitation program significantly reduced incidence of delirium, with an OR of 0.56 (95% CI 0.32–0.98; P = 0.043). Significantly more serious complications (Clavien-Dindo III-V) were present in the prehabilitation group (14.2% vs 8.9%; P = 0.036), mostly attributable to anastomotic leakages in CRC patients (7.1% in the prehabilitation group versus 2.8% in control group; P = 0.025). These complications led to a significantly higher mortality rate in this same group of patients (P = 0.034). After adjusted regression analysis however, the prehabilitation program had no significant effects on all other postsurgical outcomes. Short-term outcomes per diagnosis are added to this paper as a supplement (S3 Table).

**Table 2:** Postsurgical outcomes of control group versus prehabilitation group in all patients (N = 627).

	Total N = 627 (%)	Control N = 360 (%)	Prehabilitation N = 267 (%)	P-value
**Delirium**				
Incidence of delirium	64 (10.2)	42 (11.7)	22 (8.2)	0.16
Duration of delirium in days, median (IQR)	3 (2–5.75)	3 (2–6.5)	3 (1–4)	0.39
**Complications**				
Any complication other than delirium	242 (38.6)	133 (36.9)	109 (40.8)	0.32
Clavien-Dindo I-II	172 (27.4)	101 (28.1)	71 (27.4)	0.69
Clavien-Dindo III-V	70 (11.2)	32 (8.9)	38 (14.2)	0.036
**Length of stay**				
Length of hospital stay in days, median (IQR)	6 (4–10)	7 (5–10)	6 (4–10)	0.003
Unplanned ICU admission	57 (9.1)	27 (7.5)	30 (11.2)	0.11
ICU length of stay in days, median (IQR)	3 (1–7)	2 (1–7)	4 (2–7)	0.23
**Readmission**				
30-day readmission	44 (7.0)	22 (6.1)	22 (8.4)	0.28
**Mortality**				
During admission	20 (3.2)	9 (2.5)	11 (4.1)	0.25
30-day mortality	17 (2.7)	7 (1.9)	10 (3.7)	0.17
**Discharge dislocation**				
Discharge to new location	50 (8.0)	24 (6.7)	26 (9.9)	0.14
Discharge home with care	168 (26.8)	96 (26.7)	72 (27.4)	0.66
Discharge home without care	382 (60.9)	226 (62.8)	156 (58.4)	
Discharge to nursing home	57 (9.1)	29 (8.1)	28 (10.6)	0.26

**Table 3:** Unadjusted and adjusted regression analysis on postsurgical outcomes: Controls (n = 360) versus prehabilitation (n = 267).

	Unadjusted effects Odds Ratio (95% CI)	Adjusted effects Odds Ratio (95% CI)	P-value
**Primary outcome**			
Incidence of delirium	0.68 (0.40–1.17)	0.56 (0.32–0.98)^a^	0.043
**Secondary outcomes**			
Any complication	1.09 (0.79–1.50)	1.12 (0.80–1.57)^b^	0.52
Unplanned ICU admission	1.56 (0.90–2.70)	1.54 (0.86–2.75)^b^	0.14
30-day readmission	1.41 (0.76–2.60)	1.42 (0.75–2.68)^b^	0.29
Mortality (admission or <30 days postoperative)	1.84 (0.76–4.42)	1.50 (0.61–3.72)^b^	0.38
Discharge to new living situation	1.54 (0.86–2.75)	1.57 (0.84–2.96)^b^	0.16
	**Unadjusted effects**	**Adjusted effects**	
	**Coefficient (95% CI)**	**Coefficient (95% CI)**	
**Secondary outcomes**			
Length of hospital stay in days, B-coefficient (95% CI)	-1.3 (-3.1–0.57)	- 0.89 (-2.7–0.99)^b^	0.35
ICU length of stay in days, B-coefficient (95% CI)	-4.4 (-14.5–5.8)	- 5.7 (-16.6–5.3)^b^	0.30

a: Corrected for: Age, history of delirium, ASA ≥3 and diagnosis, based on previous research

b: Corrected for: Age, ASA ≥3, type of surgery and diagnosis

**Fig 2:**
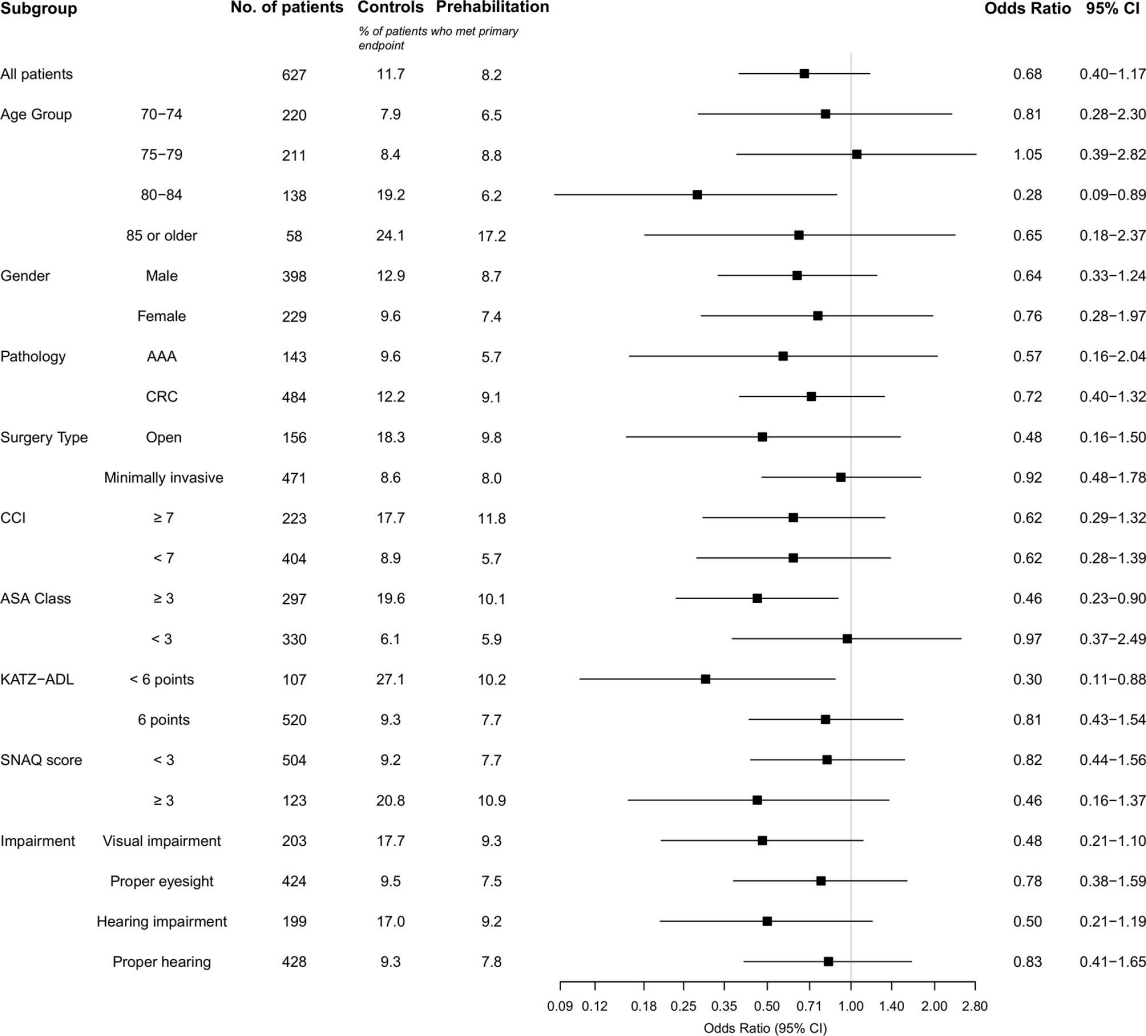
Subgroup analysis on the incidence of delirium. Odds ratios presented are unadjusted.

A subgroup analyses for the effect of the prehabilitation program on incidence of delirium is shown in Fig 2. This subgroup analysis was performed as an exploratory analysis and results are to be interpreted with caution. A significant decrease in the incidence of delirium was seen for physically impaired patients (OR 0.30; 95% CI 0.11–0.88) and in 80 to 84 year old patients (OR 0.28; 95% CI 0.09–0.89).

## Discussion

The incidence of delirium in elderly patients undergoing elective surgery for CRC or AAA can be reduced by implementing a multimodal prehabilitation program. Although prehabilitation has previously been investigated in other surgical areas (i.e. in orthopedic surgery), this study is the first to specifically investigate the role of prehabilitation in reducing the incidence of delirium after major abdominal surgery. Patients were preoperatively assessed for frailty, physical dependence, malnourishment, cognitive impairment and other factors that increase the chance of developing a postoperative delirium and were given home-based training exercises, dietary advice and nutritional support, and intravenous iron injections in case of anaemia.

After implementation of the prehabilitation protocol, incidence of delirium decreased with almost a third, which makes prehabilitation a valuable addition to already existing delirium prevention protocols. This additional advantage is achievable by a program with a relatively low burden for the patient and at relatively low costs, since exercises are home-based and the number of extra visits to the hospital is kept relatively low.

The multifactorial etiology of delirium might be an explanation for the significant reduction in delirium, while the intervention had no effect on all other postoperative outcomes. This intervention tackled multiple risk factors for delirium at once; therefore a combined effect resulting in a decrease in delirium was to be expected. The combined effect of the interventions in this prehabilitation program was not able to lower the other postoperative outcomes; outcomes which have risk factors, (e.g. type of surgery and intraoperative factors) that cannot be improved by a prehabilitation program. This is in line with recent systematic reviews on the effectiveness of multimodal prehabilitation in colorectal cancer patients. They concluded that no multimodal prehabilitation study, that included physical exercises, nutritional intervention and anxiety reduction, was able to reduce the number of postoperative complications or reduce length of hospital stay [35, 56, 57]. One of these reviews showed two unimodal programs on prehabilitation in colorectal cancer patients, one involving supervised high-intensity exercise training [58] and one involving preoperative nutritional support [59], that did improve these clinical outcomes. It also showed one unimodal program in AAA patients, involving supervised exercise training [60], that did improve these clinical outcomes. The final conclusion of this review however, similar to above-mentioned reviews, was that there was insufficient data to recommend routine clinical implementation of either unimodal or multimodal prehabilitation programs due to substantial heterogeneity [37].

The current study showed an increase in severe complications in CRC patients, which the authors feel is unlikely to be attributable to the prehabilitation program. This effect on complications may be explained by the bigger number of physically impaired patients and the higher burden of comorbidity that was found in the prehabilitation group, since this effect was nullified after correction. In contrast, the lower rate of open surgery might have compensated for these factors.

The compliance rate in the current study was almost 75%, which was just a little less compared to the highest compliance rates of unsupervised exercises in other studies [35, 56, 57]. Supervised exercise programs reach a compliance rate of almost 100%; however, this does not result in better postoperative outcomes [35, 56, 57]. The authors hypothesized that fewer people would refuse to participate in the program due to the home-based setting of the program and expected the compliance rate to be higher due to a lower patient burden. The main reasons patients provided for non-compliance were that their physical condition was good enough, that exercises were too time-consuming or that they just did not feel like exercising.

The mean length of prehabilitation was just over five weeks, comparable to past prehabilitation studies [35, 56, 57]. A longer time of prehabilitation would possibly improve results, however due to quality guidelines, the time from diagnosis to surgery may not exceed six weeks [61]. In contrast to these quality guidelines, two recent studies found no association between delay and rates of readmission, reoperation and mortality [62, 63]. Future studies could therefore try to achieve better results by extending the prehabilitation period.

Sample sizes of previous studies investigating the effect of their prehabilitation program were relatively small, often not exceeding 100 patients in total. These studies often involved much younger patients, with mean ages not exceeding 70 [35, 56, 57]. In the current study, a much larger group of patients could be included due to the design of this research. Only patients older than 70 were included in the prehabilitation program, since improving postoperative outcomes is especially important in patients who are most at risk of adverse outcomes [13]. Moreover, these patients are most likely to achieve meaningful improvement of their physical condition [64].

### Limitations

The risk of bias in this study was relatively high. Randomization, allocation concealment and blinding were not possible due to the design of this study. The outcome assessors were unaware of trial participation and used the same standardized method for diagnosing delirium as used in non-study patients; however, the electronic patient file does show a patients’ enrollment in medical research. The outcome assessors were aware of the intervention that was introduced and could therefore have been aware of a patient being included in the study, making this an important potential source of bias. The risk of bias was partially compensated by performing this trial in a single center, by letting the retrospective period connect directly to the prospective period and by letting the same group of surgeons and hospital staff provide patient care. During the time of this research, no significant changes have been made in diagnostic method or treatment protocols. Additionally, by applying the same set of inclusion and exclusion criteria the control and prehabilitation group were expected to be similar. Despite these similarities in care, by using an uncontrolled design and not using a concurrent control group, this still remains a potential source of bias.

Another limitation of this study is including both CRC and AAA in the current study. While both conditions can be and have been used together in the definition of major abdominal surgery, they are fairly different. To illustrate: surgery for these diseases is carried out by surgeons from different disciplines (e.g. colorectal and vascular surgeons).

Finally, prehabilitation is limited by the fact that those that partake in the programs are generally more motivated (and possibly fitter) than those who do not.

### Future research

Based on experience from this study and feedback from patients, the biggest challenge for future prehabilitation programs is adequately providing patients with information. Crucial to a better compliance is providing patients with sufficient and understandable information, emphasizing the importance of these programs and providing patients with custom-made exercises fitting an individual patient’s needs. By doing so, patient and caregiver can together engineer a tailored program through shared decision making and thereby increase compliance and, hopefully, decrease postoperative adverse events. Results of this study can aid in accomplishing a better understanding of the importance of prehabilitation.

Imperative in developing successful prehabilitation programs is better stratification and identification of patients who are most at risk for developing postoperative complications. Specific risk factors of frailty, most likely to improve through prehabilitation, should be identified and tackled per patient individually, in order to create a program specifically helpful for that individual patient.

In future research investigating this or new prehabilitation programs, it is advisable to assess the effectiveness of the program by building in measurements to assess improvements in the different components of the program.

The results of this study are promising, but do not provide conclusive evidence due to flaws in the design of this study. Future studies should use a higher quality study design, for example a controlled before-and-after study or a (cluster) randomized controlled trial.

## Conclusions

In elderly patients undergoing elective major abdominal surgery, the incidence of postoperative delirium can be reduced by implementing a prehabilitation program, during the pre-admission period, which focuses on optimizing patients’ fitness and nutritional status, and tackling factors of frailty and anaemia. Reducing delirium in these patients is feasible and safe with an easy-to-perform program. This program did not reduce LOS, the number and length of unplanned ICU admissions, and rates of other postoperative complications, readmissions, institutionalization and short-term mortality.

## Supporting information

S1 Table Complete overview of the study period. (DOCX)

S2 Table Full sample baseline comorbidities and baseline comorbidities per diagnosis in control group and prehabilitation group. (DOCX)

S3 Table S3 Postsurgical outcomes of control group versus prehabilitation group in AAA (N = 143) and CRC (N = 484) patients. (DOCX)

S1 File TREND checklist. (PDF)

S2 File Original protocol. (PDF)
