# Supplementary material for: Multimodal prehabilitation to reduce the incidence of delirium and other adverse events in elderly patients undergoing elective major abdominal surgery: An uncontrolled before-and-after study
Source: PLoS One. 2019 Jun 13;14(6):e0218152. doi: 10.1371/journal.pone.0218152 (PMC6564537; doi:10.1371/journal.pone.0218152)
Supplement: S1 Table — (DOCX) [file pone.0218152.s001.docx]

| **Supplement 1. Complete overview of the study period.** | | | | | | | | | | |
| --- | --- | --- | --- | --- | --- | --- | --- | --- | --- | --- |
|  | | **Study period** | | | | | | | | |
|  |  | **Eligibility assessment** | | **Trial enrollment** | **70PLUS outpatient clinic** | **Admission** | **Discharge** | **Discharge + 2 weeks** | **6-months follow-up** | **12-months follow-up** |
| Time point | | Pathology result/ CTA | Multi-disciplinary meeting | T0 – Informed consent obtained | T1 | T2 | T3 | T3.5 | T4 | T5 |
| **Assessments:** | |  | | | | | | | | |
| Laboratory testing | |  | | | X | X | X |  |  |  |
| Nurse practitioner or investigator | Baseline patient characteristics | Pathology result to  multidisciplinary meeting: <1 week. Multidisciplinary meeting to T0: <1 week.  T0 to T1: <1 week.  T1 to admission: 10 days to 5 weeks. | | | X |  |  |  |  |  |
|  | Factors of frailty |  |  |  | X |  |  |  |  |  |
|  | MMSE |  |  |  | X |  | X |  | X | X |
|  | CCI and ASA |  |  |  | X |  |  |  |  |  |
|  | P-POSSUM |  |  |  | X |  |  |  |  |  |
|  | ISAR-HP |  |  |  | X |  |  |  | X | X |
|  | PARKER |  |  |  | X |  |  |  | X | X |
|  | SNAQ |  |  |  | X |  |  |  |  |  |
|  | KATZ-ADL |  |  |  | X |  |  |  | X | X |
|  | Caregiver burden |  |  |  | X |  |  | X | X | X |
|  | CESD-16 |  |  |  | X |  |  | X | X | X |
|  | WHOQOL-BREF |  |  |  | X |  |  | X | X | X |
| Physiotherapist | 10MWT |  | | | X |  |  |  |  |  |
|  | TCST |  |  |  | X |  |  |  |  |  |
|  | TUG |  |  |  | X |  |  |  | X | X |
|  | MIP |  |  |  | X |  |  |  |  |  |
|  |  |  |  |  |  |  |  |  |  |  |
|  | Handforce |  |  |  | X |  |  |  |  |  |
| Dietician | MNA-SF | Indications for referral to dietician: Unintentional weight loss Loss of appetite BMI < 22 Undernourishment | | | X |  |  |  |  |  |
|  | BMI |  |  |  | X |  |  |  | X | X |
|  | SNAQ |  |  |  | X |  |  |  |  |  |
| Geriatrician | Complete geriatric assessment | Indications for referral to geriatrician: Delirium in history MMSE ≤ 24 TUG ≥ 12.6 seconds Polypharmacy | | | X |  |  |  |  |  |
| **Interventions:** | |  | | | | | | | | |
| Laboratory testing | All patients | Single dose of 1000 mg Ferric carboxymaltose (Ferinject®) at day care when indicated: Hb level males < 8,1 mmol/L Hb level females <7,4 mmol/L | | | X |  |  |  |  |  |
| Physiotherapist | All patients | 30 minutes of daily walking or cycling  5 exercises to improve leg muscle strength 2x15 minutes respiratory muscle exercise Transfer training when indicated | | | X |  |  |  |  |  |
| Dietician | Malnourished patients /  MNA-SF <12 | Dietary advice on required protein and calorie intake.  Proteins: 1.2 gram/kg bodyweight (BMI<30)  Calories: WHO formula for basal need + 30%.Supplements are provided when required protein and calorie intake is not met after dietary advice. | | | X |  |  |  |  |  |
| Geriatrician | Frail patients | Non-pharmaceutical interventions to reduce risk of delirium. Pharmaceutical interventions (prophylaxis) | | | X |  |  |  |  |  |
| MMSE: Mini-Mental State Examination; CCI: Charlson Comorbidity Index; ASA: American Society of Anaesthesiology; P-POSSUM: Portsmouth Physiological and Operative Severity Score for the Enumeration of Mortality and Morbidity; ISAR-HP: Identification of Seniors At Risk – Hospitalized Patients; CES-D16: Centre for Epidemiological Studies – Depression 16 questions; WHOQOL-BREF: World Health Organisation Quality of Life – BREF; 10MWT: 10-meter Walk Test; TCST: Timed Chair Stand Test; TUG: Timed-up and Go Test; MIP: Maximum Inspiratory Pressure; MNA-SF: Mini Nutritional Assessment – Short Form; BMI: Body Mass Index; SNAQ: Short Nutritional Assessment Questionnaire | | | | | | | | | | |
