# Supplementary material for: Multimodal prehabilitation to reduce the incidence of delirium and other adverse events in elderly patients undergoing elective major abdominal surgery: An uncontrolled before-and-after study
Source: PLoS One. 2019 Jun 13;14(6):e0218152. doi: 10.1371/journal.pone.0218152 (PMC6564537; doi:10.1371/journal.pone.0218152)
Supplement: S2 Table — (DOCX) [file pone.0218152.s002.docx]

| **Supplement 2. Full sample baseline comorbidities and baseline comorbidities per diagnosis in control group and prehabilitation group** | | | | | | | | |
| --- | --- | --- | --- | --- | --- | --- | --- | --- |
|  |  | |  | | |  | |  |
|  | **Control (N = 360)** | | **Prehabilitation (N = 267)** | | | **Full sample (N= 627)** | |  |
|  | **AAA  N = 73 (%)** | **CRC**  **N = 287 (%)** | **AAA  N = 70 (%)** | | **CRC  N = 197 (%)** | **Controls N = 360 (%)** | **Prehabilitation  N = 267 (%)** | **P-value^a^** |
| **Comorbidities** |  |  |  | |  |  |  |  |
| Cardiac | 38 (52.1) | 97 (33.8) | 40 (57.1) | | 75 (38.1) | 135 (37.5) | 115 (43.1) | 0.16 |
| Pulmonary | 17 (23.3) | 66 (23.0) | 19 (27.1) | | 49 (24.9) | 83 (23.1) | 68 (25.5) | 0.49 |
| Neurologic | 25 (34.2) | 65 (22.6) | 24 (34.4) | | 57 (28.9) | 90 (25.0) | 81 (30.3) | 0.14 |
| Renal insufficiency | 22 (30.1) | 48 (16.7) | 21 (30.0) | | 39 (19.8) | 70 (19.4) | 60 (22.5) | 0.36 |
| Diabetes | 12 (16.4) | 54 (18.8) | 16 (22.9) | | 44 (22.3) | 66 (18.3) | 60 (22.5) | 0.20 |
| Hypertension | 48 (65.8) | 162 (56.4) | 50 (71.4) | | 100 (50.8) | 210 (58.3) | 150 (56.2) | 0.59 |
| Hypercholesterolemia | 40 (54.8) | 77 (26.8) | 30 (42.9) | | 47 (23.9) | 117 (32.5) | 77 (28.8) | 0.33 |
| Cognitive impairment | 3 (4.1) | 22 (7.7) | 1 (1.4) | | 18 (9.1) | 25 (6.9) | 19 (7.1) | 0.93 |
|  |  |  |  | |  |  |  |  |
| a: Calculated for full samples of control versus prehabilitation | | | |  |  |  |  |  |
