# Supplementary material for: Multimodal prehabilitation to reduce the incidence of delirium and other adverse events in elderly patients undergoing elective major abdominal surgery: An uncontrolled before-and-after study
Source: PLoS One. 2019 Jun 13;14(6):e0218152. doi: 10.1371/journal.pone.0218152 (PMC6564537; doi:10.1371/journal.pone.0218152)
Supplement: S3 Table — (DOCX) [file pone.0218152.s003.docx]

| **Supplement 3. Postsurgical outcomes of control group versus prehabilitation group in AAA (N=143) and CRC (N=484) patients.** | | | | | | | |  |
| --- | --- | --- | --- | --- | --- | --- | --- | --- |
|  | | | | | | | |  |
|  | **AAA** | | |  | **CRC** | | |  |
|  | **Control  N = 73 (%)** | **Prehabilitation**  **N = 70 (%)** | **P-value** | | **Control  N = 287 (%)** | **Prehabilitation N = 197 (%)** | **P-value** |  |
| **Delirium** |  | | | |  | | |  |
| Incidence of delirium | 7 (9.6) | 4 (5.7) | 0.39 | | 35 (12.2) | 18 (9.1) | 0.29 |  |
| Duration of delirium in days, median (IQR) | 5 (2 – 11) | 7.5 (3 – 19.5) | 0.34 | | 3 (2 – 6) | 3 (1 – 3.25) | 0.16 |  |
|  |  |  |  | |  |  |  |  |
| **Complications** |  |  |  | |  |  |  |  |
| Any complication | 25 (34.2) | 26 (37.1) | 0.72 | | 120 (41.8) | 87 (44.2) | 0.61 |  |
| Clavien-Dindo I-II | 16 (21.9) | 19 (27.1) | 0.47 | | 97 (33.8) | 56 (28.4) | 0.21 |  |
| Clavien-Dindo III-V | 9 (12.3) | 7 (10.0) | 0.66 | | 23 (8.0) | 31 (15.7) | 0.008 |  |
|  |  |  |  | |  |  |  |  |
| **Length of stay** |  |  |  | |  |  |  |  |
| Length of hospital stay in days, median (IQR) | 5 (4 – 8) | 4 (3 – 7) | 0.014 | | 7 (5 – 10) | 6 (5 – 10) | 0.072 |  |
| Unplanned ICU admission | 9 (12.3) | 10 (14.3) | 0.73 | | 18 (6.3) | 20 (10.2) | 0.12 |  |
| ICU length of stay in days, median (IQR) | 5 (1.5 – 11) | 5 (2.75 – 8.25) | 0.90 | | 1.5 (1 – 4.25) | 2.5 (1.25 – 7) | 0.23 |  |
|  |  | | | |  | | |  |
| **Readmission** |  | | | |  | | |  |
| 30-day readmission | 3 (4.1) | 8 (11.4) | 0.10 | | 19 (6.7) | 14 (7.2) | 0.84 |  |
|  |  |  |  | |  |  |  |  |
| **Mortality** |  |  |  | |  |  |  |  |
| During admission | 5 (6.8) | 2 (2.9) | 0.44 | | 4 (1.4) | 9 (4.6) | 0.034 |  |
| 30-day mortality | 4 (5.5) | 3 (4.3) | 1.00 | | 3 (1.0) | 7 (3.6) | 0.099 |  |
|  |  | | | |  | | |  |
| **Discharge dislocation** |  | | | |  | | |  |
| Discharge to new location | 2 (2.9) | 6 (8.8) | 0.27 | | 22 (7.8) | 20 (10.6) | 0.29 |  |
| Discharge to previous living location | 66 (97.1) | 62 (91.2) |  | |  |  |  |  |
|  |  |  |  | |  |  |  |  |
| Discharge home with care | 8 (11.0) | 13 (18.6) | 0.16 | | 88 (30.7) | 59 (29.9) | 0.84 |  |
| Discharge home without care | 58 (79.5) | 48 (68.6) |  | | 168 (58.5) | 108 (54.8) |  |  |
| Discharge to nursing home | 2 (2.7) | 7 (10.0) | 0.17 | | 27 (9.4) | 21 (10.7) | 0.57 |  |
|  |  | | | | | | |  |
