# Supplementary material for: Multimodal prehabilitation to reduce the incidence of delirium and other adverse events in elderly patients undergoing elective major abdominal surgery: An uncontrolled before-and-after study
Source: PLoS One. 2019 Jun 13;14(6):e0218152. doi: 10.1371/journal.pone.0218152 (PMC6564537; doi:10.1371/journal.pone.0218152)
Supplement: S2 File — (PDF) [file pone.0218152.s005.pdf]

RESEARCH PROTOCOL

OUTO Trial

(Dutch: Optimalisatie oUderen Traject pre-Operatief)

PROTOCOL TITLE **: Optimalisatie Ouderen Traject Pre- Operatief**

**Short title**	**OUTO**
**Version**	**2**
**Date**	**10 February 2016**
**Coordinating investigator/project leader**	***L. Van der Laan***<br>***Molengracht 21***<br>***Lvanderlaan@amphia.nl***<br>***Phone: 076 5954033***
**Principal investigator(s) (in Dutch: hoofdonderzoeker/ uitvoerder)**	***Amphia Hospital Breda:***<br>**L. van der Laan MD PhD (department of surgery)**<br>***Co–investigators Amphia Hospital Breda:***<br>**Mw. C.A. Mosk MD**<br>**J. Wijsman MD PhD**<br>**R. Crolla MD PhD**<br>**G. Ho MD PhD**<br>**E. Veen MD PhD**<br>**Mw. D. Wielders MANP**<br>**Mw. C. van Hoof MANP**
**Sponsor (in Dutch: verrichter/opdrachtgever)**	***L. van der Laan MD PhD (department of surgery)***<br>***Amphia Hospital Breda***
**Subsidising party**	***- “ Unrestricted grant ” by Amphia Fund for Innovation***<br>***- Vifor Pharma***
**Independent expert (s)**	***J.R. Versteylen MD, Radiologist***<br>***Amphia Hospital Breda***

**Laboratory sites**	***NA***
**Pharmacy**	***NA***

PROTOCOL SIGNATURE SHEET

**Name**	**Signature**	**Date**
**Sponsor or legal representative and Principal Investigator**	**L. Van der Laan MD PhD, member of the department of Surgery, Amphia hospital Breda.**	**30-11-2015**
**Coordinating Investigator:**	**C.A. Mosk MD, Researcher, at the surgical department, Amphia Hospital Breda.**	**30-11-2015**

Optimalisatie oUderen Traject pre –Operatief (OUTO)

TABLE OF CONTENTS

1. INTRODUCTION AND RATIONALE 9
2. OBJECTIVES 10
3. STUDY DESIGN 10
4. STUDY POPULATION 12
4.1 Population (base) 12
4.2 Inclusion criteria 12
4.3 Exclusion criteria 13
4.4 Sample size calculation 13
5. TREATMENT OF SUBJECTS 14
5.1 Investigational product/treatment 14
5.2 Use of co-intervention 14
5.3 Escape medication 14
6. METHODS 15
6.1 Study parameters/endpoints 15
6.1.1 Main study parameter/endpoint 15
6.1.2 Secondary study parameters/endpoints 15
6.1.3 Other study parameters 15
6.2 Randomisation, blinding and treatment allocation .....
6.3 Study procedures 16
6.4 Withdrawal of individual subjects 18
6.4.1 Specific criteria for withdrawal 18
6.5 Replacement of individual subjects after withdrawal 18
6.6 Follow-up of subjects withdrawn from treatment 19
6.7 Premature termination of the study 19
7. SAFETY REPORTING 19
7.2 AEs, SAEs and SUSARs 19
7.2.1 Adverse events (AEs) 19
7.2.2 Serious adverse events (SAEs) 19
7.2.3 Suspected unexpected serious adverse reactions (SUSARs) .....
8. STATISTICAL ANALYSIS 20
8.1 Primary study parameter(s) 20
8.2 Secondary study parameter(s) 20
8.3 Other study parameters 20
8.4 Interim analysis (if applicable) 20
9. ETHICAL CONSIDERATIONS 21
9.1 Regulation statement 21
9.2 Recruitment and consent 21
9.3 Benefits and risks assessment, group relatedness 21

9.4 Compensation for injury 21
10. ADMINISTRATIVE ASPECTS, MONITORING AND PUBLICATION 22
10.1 Handling and storage of data and documents 22
10.2 Amendments 22
10.3 Annual progress report 22
10.4 End of study report 22
10.5 Public disclosure and publication policy 22
11. REFERENCES 23

LIST OF ABBREVIATIONS AND RELEVANT DEFINITIONS

**ABR**	**ABR form, General Assessment and Registration form. This is the application form that is required for submission to the accredited Ethics Committee (In Dutch, ABR = Algemene Beoordeling en Registratie)**
**AE**	**Adverse Event**
**AR**	**Adverse Reaction**
**CA**	**Competent Authority**
**CCMO**	**Central Committee on Research Involving Human Subjects; in Dutch: Centrale Commissie Mensgebonden Onderzoek**
**CV**	**Curriculum Vitae**
**EU**	**European Union**
**GCP**	**Good Clinical Practice**
**IB**	**Investigator's Brochure**
**IC**	**Informed Consent**
**METC**	**Medical research ethics committee (MREC); in Dutch: medisch ethische toetsing commissie (METC)**
**SAE**	**Serious Adverse Event**
**Sponsor**	**The sponsor is the party that commissions the organisation or performance of the research, for example a pharmaceutical company, academic hospital, scientific organisation or investigator. A party that provides funding for a study but does not commission it is not regarded as the sponsor, but referred to as a subsidising party.**
**Wbp**	**Personal Data Protection Act (in Dutch: Wet Bescherming Persoonsgevens)**
**WMO**	**Medical Research Involving Human Subjects Act (in Dutch: Wet Medisch-wetenschappelijk Onderzoek met Mensen**
**LLA**	**L. Van der Laan MD, PhD.**
**VNP**	**Nurse practitioner specialised in vascular surgery (in Dutch: verpleegkundig specialist vaat)**
**CM**	**Mw C. A. Mosk MD**
**EVAR**	**EndoVascular Aneurysm Repair**
**MMSE**	**Minimal mental state exam**
**MNA**	**Minimal nutritional assessment test**
**WHOQOL**	**World health organisation Quality of Life,**

Optimalisatie oUderen Traject pre –Operatief (OUTO)

SUMMARY

Rationale: Due to a growing elderly population, the demand for health care is expected to increase. Elderly are prone to have a higher incidence of delirium, which results in a longer hospital stay, more complications, and a higher rate of mortality.

Objective: Our aim is to reduce the incidence of delirium in patients over 70 from 15% to 7.5 %. Our secondary aim is to reduce length of hospital stay, number of complications and mortality.

Study design: This study is an observational interventional study.

Study population: All men and women aged 70 or older who will undergo elective surgery, for either colorectal cancer or abdominal aortic aneurysm are included in this study. Both laparoscopic (including EVAR) and open procedure are investigated.

Intervention (if applicable): Within one week after confirming the indication for surgery and five weeks before surgery patients will visit the 70 PLUS outpatient clinic in our surgery department. We aim to investigate all factors related to frailty. A dietician and a physiotherapist will provide instructions to apply at home. If anaemic, an intravenous iron injection will be given. A geriatrician will be consulted if the patient has a high chance of developing a delirium during admission.

Main study parameters/endpoints:
Incidence of delirium is our primary outcome. Secondary outcomes include duration of hospital stay and six month mortality. We will investigate aspects of frailty that are related to the incidence of delirium.

Nature and extent of the burden and risks associated with participation, benefit and group relatedness:
Patients will be asked to answer questionnaires at the outpatient clinic, during admission, and at 6 and 12 months after surgery. At the outpatient clinic blood will be drawn, which is repeated at admission and on the day of discharge. Also physiotherapists will examine the physical condition. A personalised exercise program will be made for each patient. These exercises must be done daily and a diary must be kept.

1. INTRODUCTION AND RATIONALE

The population of people above 65 years old is expected to double in the next 35 years. The increase of age is associated with an increase in need for medical care and number of patients requiring surgery. A higher rate of post–operative complications, like a delirium (37%), longer hospital stay, ICU admission, and mortality is associated with increasing age.[1, 2]
In our hospital, within the population of 70 years or older, approximately 85 patients per year undergo surgery for colorectal cancer and about 50 patients per year undergo surgery for abdominal aortic aneurysm. We expect an increase in the number these surgical procedures performed during the coming years (also due to the implementation of screening programs).

A delirium is often a fatal disorder. It affects as much as 50 % of the elderly people who are hospitalised. In the population described above, the incidence of a delirium is about 15%. The 6-month mortality is increased within patients with a postoperative delirium (20%) versus no delirium (3%). [3]
In short a delirium is a common complication after surgical intervention and results in a significant decrease in quality of life, an increased rate of complications, mortality and additionally is associated with high costs. Therefore a delirium holds essential health relevance.

The cause of a delirium can be the result of a single factor but is mostly a combination of different factors at once. Many factors have been identified and validated to be an independent cause of a delirium. These factors of frailty are for example the patient's nutrional state, physical health, the use of medication, the patient's mental state, laboratory results (such as anaemia and renal function), delirium in patient's history, visual and hearing impairment.
At the moment physicians are already more aware of these frail elderly and these factors of frailty but it is only tackled at admission in hospital or at long care facilities. A delirium is preventable in 30 to 40 % of the cases. Therefore the Hospital Elder Life Program (HELP), a worldwide non –pharmaceutical program, is implemented. This program starts at admission and is proven to be effective to prevent a delirium. [3-5] Another recent study, for patients with an acute hip surgery, shows that specific ortho – geriatric care during admission improves the outcome 4 months after surgery. [6]
We believe that these elements of frailty can be approached and tackled before admission in case of elective planned surgery. So improvement of patient's nutrional state, patient's physical health or deviant laboratory results can be achieved.

The goal of this study is to anticipate on these factors of frailty in an early phase of care to decrease the incidence of a delirium. We will optimise patients during 5 weeks before their surgery, with the intention to minimise the incidence of delirium, complications, hospital stay and mortality. Both a non–pharmaceutical as a pharmaceutical approach is implemented in this program.

2. OBJECTIVES

Our primary aim is to address and tackle the factors of frailty in an early phase, which is possible in elective planned surgery.
We hypothesise that *optimal* preparation, starting 5 weeks before surgery, will reduce the incidence of delirium from 15% to 7,5%. [7]
Our secondary aim is to reduce post-operative complications, hospital stay, and the 6 months mortality compared to a retrospective database made.
We will analyse all pre-, peri -and postoperative factors and the effect on the incidence of delirium, other complications and mortality. This will be done to create a risk model for patients to assess the risk for adverse events post-operatively. Also the costs and cost–effectiveness of this program will be investigated.

In addition we will observe the experience of the first caregiver during the course of illness of the patient. More often direct family and friends are asked to help in the support of patients in need in all forms of care in the Netherlands. First caregivers are asked to actively support the patient in this program. Caregivers are often direct family, partner or children of who their social life and daily activities can be disturbed due to this program.

3. STUDY DESIGN

This study will be a prospective interventional study, starting 1 January 2016, and planned until 31 December 2017.

A pre–operative clinical pathway is set up for patients of seventy years or older with surgical indication for colorectal cancer or abdominal aortic aneurysm, the 70 PLUS outpatient clinic in our surgery department. Within one week since indication for surgery is decided, a nurse practitioner, a physiotherapist and a dietician will see these patients and all factors of frailty will be investigated. If needed, a geriatrician will also see the patient for complete geriatric assessment and advice during admission to prevent delirium (criteria for CGA is described in *chapter 6.2 study procedures*). Diet advice and physical exercises will be given to all patients in need. Patients must follow these advices and keep a diary.
All patients aged ≥ 70 years will be included in this study to follow our pre–operative clinical pathway for optimisation. Also patients, who are not clinically fit to wait for 5 weeks will be included and optimised until surgery.
We propose an optimum preparation time during 5 to 6 weeks till surgery. This is based on the SONCOS quality guidelines 2015 for colorectal cancer, in which a maximum delay of 6 weeks is proposed from pathology result until surgery (Addendum: SONCOS quality guidelines 2015 for colorectal cancer).

Prospectively gathered data of all cases will be compared to a retrospective, historical, control group of comparable patients from 1 March 2013 till 1 March 2015.
These patients have not been evaluated for factors of frailty in advance. This is a cohort of patients of the age ≥70 years who received treatment for colorectal cancer with or without neo-adjuvant therapy (radiation or chemotherapy). Both open and laparoscopic surgery will be included.
Also patient who received treatment for abdominal aneurysm of the age ≥70 are included. These patients underwent an EVAR or open repair. (Complete description of the pathway in the retrospective group is described in *chapter 6.2 Study procedure* and addendum *Amphia protocol Colonchirurgie*)
Only electively planned surgery is included, all emergent or urgent surgical procedures are excluded.
Data of patients in both groups will be gathered in the Amphia Hospital Breda, the Netherlands.
All information will be gathered using Hyperspace Version IU4 (Epic, Inc., Verona, WI).

The historical patient group is based on patients admitted to the Amphia Hospital Breda in 2013 till November 2015. All data is gathered retrospectively but due to the use of the electronic patient file (Hyperspace Version IU4 (Epic, Inc., Verona, WI)) data was saved prospectively. No recent major changes have been made to indicate new procedures or pathways for the interventional care group compared to the historical patient group. The historical group will be comparable to the interventional care group in age (≥ 70 years), ethnicity, gender and indication for surgery. Patients with emergent or urgent surgical intervention won't be included in this study, neither in the historical cohort. All patient who underwent neo adjuvant chemo – and / or radiotherapy are included.
However no questionnaires are undertaken in the historical group, hence the lack of prospective knowledge concerning dementia (scored by MMSE), quality of life or depression scale.

4. STUDY POPULATION

4.1 Population (base)

All patients with gastro –intestinal carcinoma, who are diagnosed in the Amphia Hospital, Breda (clinically or outpatient clinic), will be discussed during a multidisciplinary meeting with gastroenterologist, GI- surgeon, radiologist and pathologist. Final treatment agreement and advice (if patient will be operated on) is made during this meeting.
All patients with aortic abdominal aneurysm, who are diagnosed in the Amphia Hospital, Breda (clinically or outpatient clinic), will be discussed during a multidisciplinary meeting with vascular surgeons and radiologist. Final agreement, if operation is needed, is made during this meeting.

During these meetings patients are selected to be included in our study. Next, either a gastro-enterologist or vascular surgeon will give written and spoken explanation about the study to obtain informed consent.

All patients of 70 years or older will be included in this study and seen at the 70 PLUS outpatient clinic. Both patients fit to wait for 5 weeks until surgery and patients who must be electively planned within 5 weeks will follow the pre–operative clinical pathway. (see figure).
This is done to obtain the best comparable population, in comparison with the retrospective population in which all patients are included with elective planned surgery of ≥ 70 years old

Flowchart Inclusion of patients

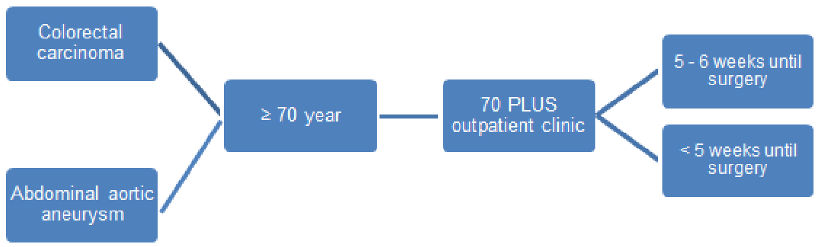

We assume to be able to include 275 patients over a period of two years, starting 1 January 2016 until 31 December 2017. About 20% of these patients will be included for an abdominal aortic aneurysm and 80% for gastro –intestinal carcinoma. [1]

Inclusion criteria

In order to be eligible to participate in this study, a subject must meet all of the following criteria:

- all patients of 70 or older;
- all Ethnicities;
- male or female;
- patients who will be operated on due to colorectal cancer or abdominal aortic aneurysm;
- only primary surgery, or patients who had abdominal surgery more than one year ago;
- the surgical procedure can be laparoscopic, open or EVAR;
- the surgical procedure can be curative or palliative;
- patient or attorney –in –fact are capable to give written informed consent.

Exclusion criteria

A potential subject who meets any of the following criteria will be excluded from participation in this study:

- all patients with moderate to severe cognitive impairment or dementia in medical history;
- emergent surgery (clinical conditions that mandate a surgery within 12 hours of admission);
- urgent surgery (clinical conditions that mandate surgery between 12 and 72 hours of admission);
- patients not willing to participate or to fill in the questionnaires;
- all patient who do not speak Dutch or English.

Demented patients

Demented patients are included as a subgroup. Demented patients are important to optimise due to their fragile state, functional dependency and poor nutritional status.

This patient group will undergo the same treatment as non-demented patients. However, we will take into account the capability of the patient to answer questionnaires and undergo the exercises. This will all be done in firm consultation with the first care giver. Informed consent is asked to the patient and first care giver.

However, severe demented patients are rarely operated on in case of AAA or CRC. The risk in these patients in elective setting is very high compared to the benefits, also these patient usually start having complaints in worse case and are often operated on in acute setting (which is an exclusion criterion in this study).

Sample size calculation

To reduce the percentage of delirium form 15% to 7,5%, with a power of 80% with 5% two–sided significance level, a 50 -50 trial, needs 550 patients, or 275 patient per study arm.

We propose to make a comparison to a retrospective control group of patients studied between 2012 and 2014 in the Amphia Hospital, Breda. [1]

We aim to include 275 patients in the prospective study, which is feasible during approximately 2 years of accrual.

5. TREATMENT OF SUBJECTS

This prospective study is set up as an outpatient clinical pathway in which we intend to intervene in the usual pathway until operation. Within one week after setting indication for surgery, patients will be assessed. A nurse practitioner, a dietician and a physiotherapist will see the patients and if necessary a geriatrician will be involved (specific criteria for consulting dietician or geriatrician are defined in chapter *6.2 Study procedures*).
In the usual pathway, patients are seen by the surgeon and are planned for surgery as soon as possible (which is the case in the retrospective group).

Investigational product/treatment

Laboratory results to evaluate haemoglobin for intravenous iron (IVI), electrolytes for suppletion and nutritional status for refeeding.
Dietician's advice or refeeding products in patients with malnutrition.
Physical exercise for all patients and keeping a daily diary.
In case a geriatrician is consulted, they will assess if preventive interventions for delirium are needed (e.g. starting haloperidol).

Use of co-intervention

Intra venous Iron–injection (Vifor Pharma) will be given in all anaemic patients, similar to the usual protocol for anaemic patients. (Addendum)
Anaemia was defined as a haemoglobin level of < 7.4 mmol/L(<120 g/L) for women and < 8.1 mmol/L for men (<130 g/L). In addition the WHO defines severe anaemia as a haemoglobin of 4,4 mmol /L (<70g/L) and very severe anaemia as a haemoglobin of 2,5 mmol/L (<40 g/L). [8] [9]
If anaemic patients are using oral medication for iron deficit, they should stop this medication one day before starting intra venous iron –injection. Addendum: protocol Iron –injection in Dutch

Escape medication

Escape medication for intra venous iron–injection are
- Adrenaline (1mg, 1ml) subcutaneously;
- Tavegyl (1 mg) oral or Tavegyl (2 mg) intra-venous;
- Dexamethason (4 mg, 1 ml) intra-muscular.

6. METHODS

6.1 Study parameters/endpoints

Main study parameter/endpoint

Our main parameter is the incidence of a delirium is screened by DOS scores and confirmed by DSM IV classification. [10] Duration in days of the delirium will be investigated.
Our main endpoints are mortality within one year and follow up till 12 months after surgery.

Secondary study parameters/endpoints (if applicable)

Secondary we will measure complication rate, hospital stay, ICU admission, and ICU duration of stay.

Other study parameters (if applicable)

At the moment of inclusion, all patient characteristics will be investigated including all factors of frailty causing delirium. These parameters will be investigated to indicate their relation with the incidence of delirium. [1, 7, 11-13]

6.2 Study procedures

Standard Care

Standard pathway until surgery contains a visit to the outpatient clinic of either a gastroenterologist or a vascular surgeon to diagnose patients' condition. Patients will be discussed during multidisciplinary meeting, where the decision to operate is made. Next patient will be seen by their surgeon give explanation about the surgical procedure and to screen if patient is fit for surgery. Normal surgical preparations are done. At admission patient will be screened for delirium by a ward nurse. If necessary a dietician or geriatrician will visit. Standard care during admission is applied. (Addendum *Amphia protocol Colonchirurgie)*

OUTO Care

For all patients of ≥ 70 with surgical indication for colorectal cancer or abdominal aortic aneurysm, the clinical pathway 70 PLUS outpatient clinic is set up in our surgery department.
During this pathway, we will screen for delirium and actively investigate and tackle their factors of frailty. The complete assessment en study procedure is described underneath (see figure).

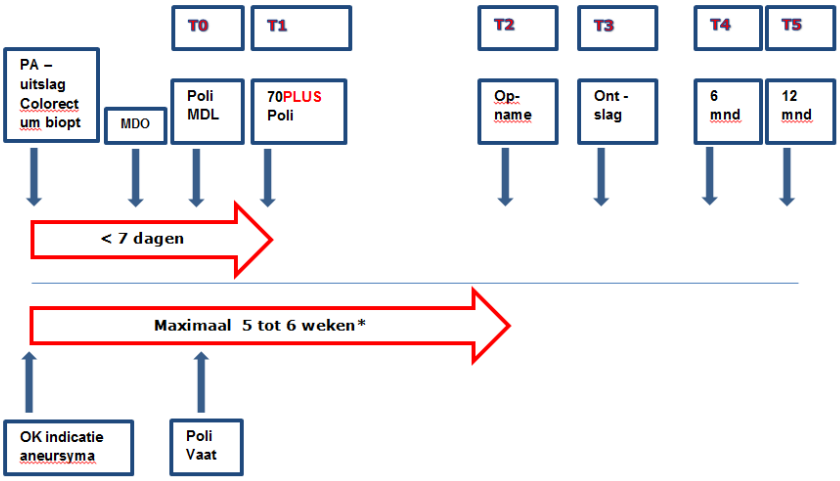

Timeline OUTO pathway.

* Quality guidelines SONCOS for colorectal carcinoma.

When patients arrive at the 70PLUS outpatient clinic (T1) a VNP will assess all base–line characteristics and predictors of delirium; age, gender, surgical history, comorbidity (cardiac, pulmonary, neurological, renal, dementia, diabetes), use of medication, smoking status, use of alcohol, social economic status, use of glasses or hearing problems, BMI, home situation and psychological history.

Burden of comorbidity is quantified using the Possum score, Charlsonn morbidity score and the American Society of Anesthesologisch score (ASA).[12, 14, 15]

The following questionnaires will be taken for cognition and mental health [16-18]:
- WHOQOL-BREF is used to determine quality of life;
- Mini mental status exam (MMSE) to assess cognitive dysfunction.

All patients will visit the physiotherapist, to assess patients' condition and strength using the following tests:

- Time Up and Go test (TUG test); [19]
- Timed chair stand test;
- The maximum inspiratory pressure (MIP) (using MicroRPM™, PT Medical);
- Gait speed using the 10 meter walking test [20];
- The hand force (using Hydraulic Hand dynamometer, JAMAR™);

The KATZ – ADL score is a standard test used in all patients admitted in our hospital to assess dependency and function at home.

Laboratory results measured during workup for diagnosis were used. If not investigated during work up additional blood will be drawn to complete our lab results.
Laboratory results used for this study are:
- haemoglobine, hematocrite;
- erythrocytes, thrombocytes;
- INR;
- Electrolytes (Sodium, potassium, chloride);
- Renal function (kreatinine, GFR, ureum);
- Liver functions (ASAT, ALAT);
- Lipid spectrum;
- Vitamine B, D and folic acid and prealbumine.

Anaemic patients (haemoglobuline level of < 7.4 mmol/L(<120 g/L) for women and < 8.1 mmol/L for men (<130 g/L)) will be given iron–injection after they filled in the WHOQOL-Bref. These patients will be admitted at day-care, which is standard care for all patients operated on with anaemia. [21] For the patients operated on in case of abdominal aortic aneurysm iron-Injection is part of this new pre – operative pathway and not former standard care.

Dietician is consulted when patient lost weight without purpose or lost appetite.
Nutrition is quantified using BMI, the minimal nutritional assessment test (MNA) and SNAQ -RC score. Also prealbumine is tested in the laboratory results.

Complete geriatric assessment is done for patients with the following criteria:
- MMSE score < 24 points;
- MMSE score < 26, If patient went to secondary school or university;
- delirium in medical history;
- polypharmacy:
    - Colorectal patients with more than 4 different kinds of medication
    - Vascular patients with more than four different kind of medication next to regular vascular preventive medication (Anti-coagulants, a statin, or bloodpressure medication)
- Time –up and Go Test (TUG Test) of ≥ 15 seconds.

Geriatrician will assess if preventive medication, like Haloperidol, is necessary.

The Care giver strain index (CSI) will be used to investigate pressure of first care giver during the course of illness of the patient.

At hospital admission (T2) patients will be seen by the same physiotherapist and researcher.
The physiotherapist will assess patients' condition and function using the TUG test, MIP, and 10 meters walk test.
The same laboratory results will be measured. The outcome of the WHOQOL–Bref can be aberrant if patients endure depressive feelings. This is why we will screen for depression, using

CES D-16 questionnaire at admission.

During admission a physician will see all patients on a daily basis. A nurse, while providing regular daily care, will screen for a delirium using the Delirium Observation Screening Score (DOSS) three times a day. DOSS is a shortened version with 13 items and is scored three times a day. A delirium is highly presumable if the patient had a DOSS of ≥ 3 and scored three times in a row. When delirium is suspected a geriatrician will be consulted to confirm the diagnosis using the DSM-IV criteria. [10]
Also nurses will daily examine the level of pain using the Visual Analogue Score (VAS).

Data about the surgical procedure will be gathered. American Society of Anaesthesiologists' (ASA) classification for physical health is determined before surgery from history and physical examination by the attending anaesthesiologist. Anaesthesia time will be calculated as the duration between tracheal in– and extubation. Also blood loss during surgery and complications during surgery will be noted.
All Patients undergoing surgery for colorectal carcinoma or abdominal aortic aneurysm surgery will receive general anaesthesia. Epidural anaesthesia is used as an adjunct as part of the fast track protocol. If patient is not eligible for epidural anaesthesia is not eligible (in case of allergies or co – agulopathy), a Patient-Controlled Analgesia pump (PCA-Pump) with Morphine will be used as an alternative . Patients operated on with EVAR will not receive an epidural or a PCA.
Other parameters gathered during admission, are complication, ICU stay, transfusion of packet cells, and mortality. Complications are classified using the Clavien –Dindo classification of surgical complications. [22]
At discharge (T3) patients will fill in WHOQOL-Bref, MMSE will be taken, the same blood will be drawn and the CSI will be filled in by first caregiver.
At follow up after six months (T4) and 12 months (T5) WHOQOL-Bref, CSI, MMSE and CESD-16 will be taken. Also function is tested using the TUG test and KATZ – ADL.

Table 2 describes when measurements are repeated to evaluate the effects of the intervention before surgery and after surgery. Laboratory results and physical exercises will be measured at admission.

Addendum describes all questionnaires, patient’s chard, and laboratory results.

6.3 Time line

This study will start 1 January 2016 and will continue until 275 patients are included. We expect to reach this amount round 1 January 2018. Data analyses (including one year follow up) are expected to be finished in 2019.

6.4 Withdrawal of individual subjects

Subjects can leave the study at any time for any reason if they wish to do so without any consequences. The investigator can decide to withdraw a subject from the study for urgent medical reasons.

Specific criteria for withdrawal

Patients will be excluded from this study when they do not want to fill in questionnaires or do not want to visit our outpatient clinic during follow up.
Patients who are excluded after surgery or after discharge will be noted as ‘lost to follow up’. They will not visit the 70PLUS outpatient clinic at 6 months or 12 months.

6.5 Replacement of individual subjects after withdrawal

Patients withdrawn from this study will not be replaced.

6.6 Follow-up of subjects withdrawn from treatment
Patients withdrawn from our study will follow usual follow up, only patient information about complications en mortality will be used.

6.7 Premature termination of the study
Study will be terminated in case of a higher complication rate or mortality compared to former research done. [1] After one year, analysis will be done to investigate if no negative effect occurs due to this study, this is done by comparing complication rate, incidence of delirium, rate of ICU admission, length of hospital stay and mortality within 6 months, with the historical patient group.
Also we will investigate the use and efficiency of iron injection in anaemic patients.
Interim analyses are not possible due to the small amount of patients gathered. No significant data can be obtained.

7 SAFETY REPORTING

7.1 Temporary halt for reasons of subject safety
In accordance to section 10, subsection 4, of the WMO, the sponsor will suspend the study if there is sufficient ground that continuation of the study will jeopardise subject health or safety. The sponsor will notify the accredited METC without undue delay of a temporary halt including the reason for such an action. The study will be suspended pending a further positive decision by the accredited METC. The investigator will take care that all subjects are kept informed.

7.2 AEs, SAEs and SUSARs

Adverse events (AEs)

Adverse events are defined as any undesirable experience occurring to a subject during the study, whether or not considered related to trial procedure. All adverse events reported spontaneously by the subject or observed by the investigator or his staff will be recorded.

Serious adverse events (SAEs)

A serious adverse event is any untoward medical occurrence or effect that
- results in death;
- is life threatening (at the time of the event);
- requires hospitalisation or prolongation of existing inpatients' hospitalisation;
- results in persistent or significant disability or incapacity;
- is a congenital anomaly or birth defect; or
- any other important medical event that did not result in any of the outcomes listed above due to medical or surgical intervention but could have been based upon appropriate judgement by the investigator.

An elective hospital admission will not be considered as a serious adverse event.

The use of i.v. iron injection, can cause allergic reaction. This product is used normally for all anaemic patients with iron deficiency with specific surgical indication. In-hospital protocol is already in use. No other or extra care will be undertaken. Addendum describes the in-hospital protocol for the use of i.v. iron injection (in Dutch).
Due to physical exercises patient can have muscle complaints, or even fall. Usual care will be given.
All events will be reported by the investigator (CM) to the sponsor (LLA). Patient will still be included in our database and will not be withdrawn from this study when an adverse event occurs.
To our knowledge no other adverse events might occur when participating in this study.

The investigator will report all SAEs to the sponsor without undue delay after obtaining knowledge of the events, without any exceptions except for the following SAE:

- If death occurs during this study due to the normal cause of illness, we will not notify this as a SAE.

The sponsor (LLA) will report the SAEs through the web portal *ToetsingOnline* to the accredited METC that approved the protocol, within 7 days of first knowledge for SAEs that result in death or are life threatening followed by a period of maximum of 8 days to complete the initial preliminary report. All other SAEs will be reported within a period of maximum 15 days after the sponsor has first knowledge of the serious adverse events.

8 STATISTICAL ANALYSIS

Standard procedure will be followed for descriptive statistics. The primary analysis for incidence of delirium will be al logistic regression model with adjustment for prognostically important covariates: age, gender, surgical history, comorbidity, (cardiac, pulmonary, neurological, renal, dementia, diabetes), use of medication, smoking status, use of alcohol, social economic status, use of glasses or hearing impairment, BMI, home situation. We will also consider he POSSUM score and Charlsonn comorbidity score. Repeated measure analysis will be applied for measurement over time during follow up. Historic and prospective patients will be selected for analysis fulfilling the same set of inclusion criteria. Missing data will be studied for specific patterns of occurrence, and multiply imputed. Statistical analysis was performed with IBM SPSS statistics software (SPSS Inc., Chicago, Illinois, USA).

8.1 Primary study parameter(s)

Our primary study parameter is the incidence of delirium. Delirium is screened with the DOS score. DOSS is a shortened version with 13 items and is scored three times a day. A delirium is highly presumable if the patient had a DOSS of ≥ 3 and scored three times in a row. When delirium is suspected a geriatrician will be consulted to confirm the diagnosis using the DSM-IV criteria.
We will describe the severity of the delirium using the DOS score (13 points is maximum). Also duration of delirium will be measured in days.

8.2 Secondary study parameter(s)

Secondary we will measure mortality within one year after surgery. Also duration of hospital stay, ICU admission, and complications during hospital stay will be investigated.

8.3 Other study parameters

All factors of frailty will be evaluated and analysis will be done to confirm association with the incidence of delirium.

8.4 Interim analysis (if applicable)

Interim analysis is not possible, because at that point, not enough patients will be gathered to obtain any significant value.

9 ETHICAL CONSIDERATIONS

9.1 Regulation statement

The study will be conducted according to the principles of the Declaration of Helsinki 64th WMA General Assembly, Fortaleza, Brazil, October 2013 and in accordance with the Medical Research Involving Human Subjects Act (WMO) and other guidelines, regulations and Acts.

9.2 Recruitment and consent

Patients are recruited during multidisciplinary meeting. Within three days patients will be informed by their doctor (gastroenterologist or vascular surgeon) to explain the agreement made during this multidisciplinary meeting. Also they will give written and spoken information, including the informed consent form.
If patient gives spoken approval to their physician, the researcher will contact the patient to give spoken explanation about the study and to plan the 70PLUS outpatient clinic appointment. During this appointment informed consent will be obtained and the informed consent form will be signed. The patient will receive a copy of the signed informed consent form.

9.3 Benefits and risks assessment, group relatedness

The burden of this study consists mostly of the visit to the outpatient clinic and several questionnaires taken.
Probable risks to the subject consist only of drawing blood, physical exercises and if anaemic i.v. iron injection. We expect a minimal risk for adverse events due to these actions. All will be done by experienced personnel.

9.4 Compensation for injury

The sponsor/investigator has a liability insurance which is in accordance with article 7 of the WMO. Insurance contract is made with MEDIRISK.
The sponsor has an insurance which is in accordance with the legal requirements in the Netherlands (Article 7 WMO). This insurance provides cover for damage to research subjects through injury or death caused by the study.
The insurance applies to the damage that becomes apparent during the study or within 4 years after the end of the study.
Patient must claim damage or injury during the course of this study or within 4 years after the end of the study at the insurance.

10. ADMINISTRATIVE ASPECTS, MONITORING AND PUBLICATION

10.1 Handling and storage of data and documents

All data will be gathered in the electronic patient file using Hyperspace Version IU4 (Epic, Inc., Verona, WI).
A specific OUTO-study folder is created in which data is only accessible to the sponsor (LLA) and researcher (CM). A code number for each patient will be used. This folder is secured and the code is safeguarded by the researcher (CM) and sponsor (LLA). Questionnaires are securely safeguarded by the researcher and locked.
VSP, dietician, physiotherapist and geriatrician are able to fill in all data in the OUTO-study folder during patient contact.
Patient's physician or practitioner is able to see patients electronic file.

10.2 Amendments

Amendments are changes made to the research after a favourable opinion by the accredited METC has been given. All amendments will be notified to the METC that gave a favourable opinion.

10.3 Annual progress report

The investigator will submit a summary of the progress of the trial to the accredited METC once a year. Information will be provided on the date of inclusion of the first subject, numbers of subjects included and numbers of subjects that have completed the trial, serious adverse events/ serious adverse reactions, other problems, and amendments.

10.4 Temporary halt and (prematurely) end of study report

The investigator or sponsor will notify the accredited METC of the end of the study within a period of 8 weeks. The end of the study is defined as the last patient's last visit.
The sponsor will notify the METC immediately of a temporary halt of the study, including the reason of such an action.
In case the study is ended prematurely, the sponsor will notify the accredited METC within 15 days, including the reasons for the premature termination.
Within one year after the end of the study, the investigator/sponsor will submit a final study report with the results of the study, including any publications/abstracts of the study, to the accredited METC.

10.5 Public disclosure and publication policy

Research protocol and all gathered data will be offered for publication to a scientific journal.

11. REFERENCES

1. Raats, J.W., et al., *Risk Factors and Outcomes for Postoperative Delirium after Major Surgery in Elderly Patients.* PLoS One, 2015. **10**(8): p. e0136071.
2. Organisation, W.H. *Ageing and health*. http://www.who.int/mediacentre/factsheets/fs404/en/ 2015 September 2015.
3. Inouye, S.K., R.G. Westendorp, and J.S. Saczynski, *Delirium in elderly people.* Lancet, 2014. **383**(9920): p. 911-22.
4. Inouye, S.K., et al., *The Hospital Elder Life Program: a model of care to prevent cognitive and functional decline in older hospitalized patients. Hospital Elder Life Program.* J Am Geriatr Soc, 2000. **48**(12): p. 1697-706.
5. Inouye, S.K., et al., *A multicomponent intervention to prevent delirium in hospitalized older patients.* N Engl J Med, 1999. **340**(9): p. 669-76.
6. Prestmo, A., et al., *Comprehensive geriatric care for patients with hip fractures: a prospective, randomised, controlled trial.* Lancet, 2015. **385**(9978): p. 1623-33.
7. Raats, J.W., et al., *Postoperative delirium in elderly after elective and acute colorectal surgery: A prospective cohort study.* Int J Surg, 2015. **18**: p. 216-9.
8. organisation, W.h., *Iron Deficiency Anaemia, Assessment, Prevention and controle. A guide for programme managers.* 2001, WHO: http://www.WHO.int/nutrition/publications/en/ida_assessment_prevention_control.pdf.
9. Desormais, I., et al., *Anemia, an independent predictive factor for amputation and mortality in patients hospitalized for peripheral artery disease.* Eur J Vasc Endovasc Surg, 2014. **48**(2): p. 202-7.
10. Schuurmans, M.J., L.M. Shortridge-Baggett, and S.A. Duursma, *The Delirium Observation Screening Scale: a screening instrument for delirium.* Res Theory Nurs Pract, 2003. **17**(1): p. 31-50.
11. Inouye, S.K., *Predisposing and precipitating factors for delirium in hospitalized older patients.* Dement Geriatr Cogn Disord, 1999. **10**(5): p. 393-400.
12. Schor, J.D., et al., *Risk factors for delirium in hospitalized elderly.* JAMA, 1992. **267**(6): p. 827-31.
13. Witlox, J., et al., *Delirium in elderly patients and the risk of postdischarge mortality, institutionalization, and dementia: a meta-analysis.* JAMA, 2010. **304**(4): p. 443-51.
14. Rahkonen, T., et al., *Delirium in the non-demented oldest old in the general population: risk factors and prognosis.* Int J Geriatr Psychiatry, 2001. **16**(4): p. 415-21.
15. Copeland, G.P., D. Jones, and M. Walters, *POSSUM: a scoring system for surgical audit.* Br J Surg, 1991. **78**(3): p. 355-60.
16. Skevington, S.M., et al., *The World Health Organization's WHOQOL-BREF quality of life assessment: psychometric properties and results of the international field trial. A report from the WHOQOL group.* Qual Life Res, 2004. **13**(2): p. 299-310.
17. *Development of the World Health Organization WHOQOL-BREF quality of life assessment. The WHOQOL Group.* Psychol Med, 1998. **28**(3): p. 551-8.
18. Kaiser, M.J., et al., *Validation of the Mini Nutritional Assessment short-form (MNA-SF): a practical tool for identification of nutritional status.* J Nutr Health Aging, 2009. **13**(9): p. 782-8.
19. Huisman, M.G., et al., *"Timed Up & Go": a screening tool for predicting 30-day morbidity in onco-geriatric surgical patients? A multicenter cohort study.* PLoS One, 2014. **9**(1): p. e86863.
20. Schoon, Y., et al., *Gait speed as a test for monitoring frailty in community-dwelling older people has the highest diagnostic value compared to step length and chair rise time.* Eur J Phys Rehabil Med, 2014. **50**(6): p. 693-701.
21. Auerbach, M., L.T. Goodnough, and A. Shander, *Iron: the new advances in therapy.* Best Pract Res Clin Anaesthesiol, 2013. **27**(1): p. 131-40.

22. Dindo, D., N. Demartines, and P.A. Clavien, *Classification of surgical complications: a new proposal with evaluation in a cohort of 6336 patients and results of a survey.* Ann Surg, 2004. **240**(2): p. 205-13.
